# Retraction: COL10A1-DDR2 axis promotes the progression of pancreatic cancer by regulating MEK/ERK signal transduction

**DOI:** 10.3389/fonc.2024.1538142

**Published:** 2024-12-10

**Authors:** Frontiers Editorial Office

The journal retracts the 2022 article cited above.

Following publication, the authors contacted the Editorial Office to request the retraction of the cited article, stating that there were errors in the figures. The findings reported in the article are no longer supported by the data. An investigation was conducted in in accordance with Frontiers’ policies that confirmed this; therefore, the article has been retracted.

This retraction was approved by the Chief Editors of Frontiers in Oncology and the Chief Executive Editor of Frontiers. The authors agree to this retraction.

